# Identification of Enteric Pathogen Reservoirs and Transmission Pathways Associated with Short Childhood Stature in the Kolkata Indian Site of the Global Enteric Multicenter Study

**DOI:** 10.3390/nu16162733

**Published:** 2024-08-16

**Authors:** Kurt Z. Long, Inong R. Gunanti, Chris Stride, Johanna Sanchez, Dipika Sur, Byomkesh Manna, Thandavarayan Ramamurthy, Suman Kanungo, James P. Nataro, Helen Powell, Anna Roose, Dilruba Nasrin, Halvor Sommerfelt, Myron Levine, Karen Kotloff

**Affiliations:** 1 Epidemiology and Public Health, Swiss Tropical and Public Health Institute, 4123 Allschwil, Switzerland; 2 Faculty of Medicine, University of Basel, Peterplatz 1, 4003 Basel, Switzerland; 3 The Child Health Research Centre, Faculty of Medicine and Biomedical Sciences, The University of Queensland, Brisbane 4101, Australia; 4 The Institute of Work Psychology, University of Sheffield, Sheffield S10 2TN, UK; c.b.stride@sheffield.ac.uk; 5 ICMR—National Institute for Research in Bacterial Infections, Kolkata 700010, India; 6 Department of Pediatrics, University of Virginia School of Medicine, Charlottesville, VA 22903, USA; 7 Department of Pediatrics, Center for Vaccine Development and Global Health, University of Maryland School of Medicine, Baltimore, MD 21201, USA; 8 Department of Medicine Center for Vaccine Development and Global Health, University of Maryland School of Medicine, Baltimore, MD 21201, USA; 9 Centre for Intervention Science in Maternal and Child Health, Centre for International Health, University of Bergen and the Norwegian Institute of Public Health, NO-5020 Bergen, Norway

**Keywords:** enteric pathogen reservoirs, transmission, child stature, rotavirus, DEC, GII mediation, India

## Abstract

Age-stratified path analyses modeled associations between enteric pathogen reservoirs, transmission pathways and height-for-age z-scores (HAZ) to identify determinants of childhood growth in the Kolkata, India site of the Global Enteric Multicenter Study (GEMS). Models tested direct associations of potential pathogen reservoirs with HAZ at 60-day follow-up in separate moderate and severe diarrhea (MSD) case and control cohorts or indirectly when mediated by enteric infections. In the MSD cohort, rotavirus and typical EPEC (tEPEC) infections among children 0–11 months of age and ST-ETEC infections among children 12–23 months of age were associated with lower HAZ. Handwashing after defecating and before cooking reduced impaired growth through reductions in rotavirus and tEPEC infections. Water storage increased rotavirus and ST-ETEC infection risks, resulting in increased impaired growth, but was reduced with reported child feces disposal. The GII norovirus variant was inversely associated with HAZ among children 12–59 months of age in the control cohort. Reported handwashing before the handling of children reduced GII infections and impaired growth. Boiling water and the disposal of children’s feces mediated by stored water were positively associated with HAZ. The targeting of pathogen-specific reservoirs and transmission pathways may more effectively improve childhood linear growth in South Asian urban communities.

## 1. Introduction

Significant reductions in the diarrheal disease burden among children under 5 years of age has occurred in low- and middle-income countries (LMICs) over the last several decades. However, this burden continues to be high, with approximately 1.7 billion diarrhea episodes and 446,000 associated deaths reported worldwide [1]. The global burden of childhood malnutrition remains considerable as well, with 153 million children estimated to be stunted in 2015 [2]. Poor growth and diarrheal disease are linked in a cycle initiated when children experience growth faltering following infections by such pathogens as *Cryptosporidium* and *Shigella* when untreated. Nutritionally deficient children, in turn, are more susceptible to severe enteric infections. Sub-Saharan Africa and South Asia have the greatest burden of this diarrhea disease–malnutrition syndrome, as well as the highest under-five mortality [3].

Evidence-based interventions involving improved water and sanitation and the promotion of hygiene behaviors (WASH) have been implemented in a number of LMICs [4]. However, systematic, and well-designed, multi-armed interventions have not produced consistent results with respect to the impact on enteric infection, diarrheal disease and child length/height [5,6,7]. These inconsistent results have led to calls for the development of transformational WASH programs that radically reduce fecal contamination and children’s exposure in the household environment [8]. To achieve these goals, such programs must identify what pathogen-specific reservoirs and transmission pathways in specific study communities are important in children’s exposure to different pathogens and subsequent linear growth impairment [9,10,11,12].

We developed a Bayesian path analysis to address the directed dependencies and causal connections of these relationships using data from the Kolkata, India site of the Global Enteric Multicenter Study (GEMS) [13,14]. This analysis estimated parameters for pathways between household risk factors, enteric pathogens, and length (for children up to two years of age) or height (for those two or older) at the 60-day follow-up of children in the case and control cohorts, treated as separate cohorts. For simplicity, in the rest of this paper, we will refer to both “length” and “height” as height. These models incorporated mediation analyses to test these hypothesized pathogen-specific transmission pathways and subsequently tested whether hygiene behaviors modified or blocked these pathways [15].

The specific aims of the study were to assess: (1) age-related associations of height-for-age z-score (HAZ) at 60-day follow-up with such household pathogen reservoir risk factors as animals in the household compound, household material construction and water sources in a cohort of children enrolled with moderate-to severe diarrhea (MSD) and separately in a cohort of children without diarrhea enrolled concomitantly as controls; (2) assess the hypothesized mediation of these reservoirs by enteric infections in these two cohorts to identify specific pathogen transmission pathways and; (3) assess how hygiene behaviors, water management and caretaker characteristics modulate the effect of pathogen reservoir and enteric infections on the child’s growth by presumably blocking or further promoting pathogen transmission. The results from these analyses can inform the design, implementation and evaluation of integrated interventions that more effectively reduce diarrhea disease burden and improve childhood growth through targeting critical points in pathogen transmission.

## 2. Materials and Methods

The children included in this study were enrolled at the Kolkata, India site of GEMS between 1 December 2007 and 3 March 2011. GEMS was a 3-year, prospective, matched case–control study of MSD in children aged 0–59 months residing in four African and three Asian countries [14]. The Kolkata site was selected for the current analysis to identify which household and community factors are associated with enteric pathogen exposure and childhood growth in an urban South Asian setting. The methods used in GEMS are described in detail elsewhere [13,14]. In brief, cases were aged < 59 months who sought care at study health centers with a new, acute MSD episode. Enrollment was stratified into three age categories (0–11 months, 12–23 months and 24–59 months). One to three control children without diarrhea in the preceding week matched to each case by age, sex, date of case enrollment, and residence were enrolled at home. The primary caretakers of the study children were interviewed at enrollment to collect clinical, demographic, and epidemiologic information, including maternal education, household size, household construction, water and sanitation facilities, and animals in the household. Information was also collected on reported child feces disposal patterns, handwashing patterns, and finally on water storage and treatment practices. Length or height-for-age Z scores (HAZ) at enrollment and at a 60-day follow-up (acceptable range 50–90 days) were derived using the median of three repeated height measurements at each visit for every child and standardized according to the WHO Multicenter Growth Reference Study [9,16]

Stool samples, collected from cases and matched controls at enrolment, were screened for viral, protozoa and bacterial agents as previously described [17]. Conventional culture techniques were used to screen for *Salmonella*, *Shigella* spp., *Campylobacter jejuni*, *Aeromonas hydrophila,* and *Vibrio cholerae*. Multiplex PCR developed for GEMS was used to identify the diarrhea *E. coli* pathotypes of enteroaggregative (EAEC), typical and atypical enteropathogenic (tEPEC: eae+ bfpA+; aEPEC: eae+ bfpA−) enterohaemorrhagic (EHEC) and heat stable—producing enterotoxigenic with or without heat labile toxin *E. coli* (ST ETEC and ST/LT ETEC)—herein designated as ST-ETEC. Commercial immunoassays were used to detect rotavirus, and adenovirus serotypes 40 and 41, while norovirus, sapovirus and astrovirus were detected using multiplex reverse transcriptase (RT) PCR. Finally, *Giardia lamblia*, *Entamoeba histolytica* and *Crytosporidium* spp. were detected using parasite-specific commercial immunoassays [14,17].

### Statistical Analyses

**Figure 1:**
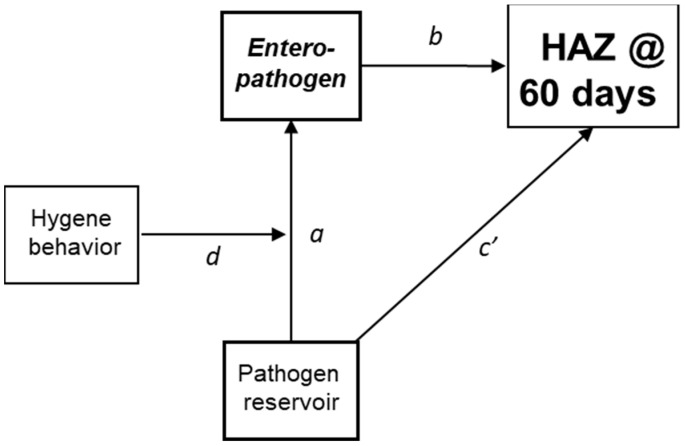
Conceptual model for enteric pathogen reservoirs, transmission pathways and change in height-for-age z-scores. a,b: pathway between pathogen-specific reservoirs and pathogen transmission and HAZ. c′: direct effect of pathogen reservoir on HAZ. d: hygiene behaviors that block transmission.

The principal outcome of the current analysis was HAZ at 60-day follow-up. The separate analyses in the MSD and in the control cohorts were stratified by the three age categories to address differences in growth, with HAZ at baseline included to control for initial differences. The separate analyses of the two cohorts was necessary due to the oversampling of children with MSD in GEMS, which if not taken into account would lead to biases. Systematic reviews and previous GEMS results have reported associations of household risk factors with pathogen infections in children, risk factor associations with reduced child stature and associations of enteric infections with reduced stature [9,12,18,19]. We hypothesized that these separate associations represent components of pathogen transmission pathways linking household reservoirs, specific pathogen infections in children and subsequent impaired child growth. Accordingly, a conceptual model was developed, which delineated how specific transmission pathways mediated associations between reservoirs to children’s exposure and growth impairment (Figure 1 pathway a–b). These pathways can be blocked or modified by hygiene behaviors which moderate these indirect effects (d in Figure 1).

Those pathogens identified as important causes of diarrhea at the Kolkata site in GEMS, i.e., rotavirus, *C. jejuni*, *S. flexneri*, *Aeromonas*, ST-ETEC, aEPEC, *Cryptosporidium* and norovirus, were considered for the model. *Giardia* was also considered, given its effect on growth [9,20]. The separate analyses carried out for the children in the MSD and control cohorts determined if transmission pathways that lead to asymptomatic enteric infections differ from pathways for pathogens that cause diarrhea [20].

This model tested whether water sources and storage, the use of unimproved sanitation and means for the disposal of child excreta, and animal and human reservoirs were potential reservoirs involved in pathogen transmission pathways. Binary variables representing reservoirs included water sources (public tap, deep tube well, shallow tube well, water piped into the yard or piped into house; no = 0, yes = 1 for each), child given stored water (no = 0, yes = 1), feces disposal facilities classified according to WHO guidelines [21] (traditional latrine or pit latrine = 0, flush toilet or water seal VIP latrine = 1), shared facilities with other households, child feces disposal patterns (disposed in environment = 0, toilet or latrine = 1), animals in the household (goat, dog, fowl, cat, or rodents; no = 0, yes = 1 for each), two or fewer children less than 5 years of age in the household vs. more than 2 or more (no = 0, yes = 1), and floor construction (earth, sand or dung = 0, tile, cement or wood = 1). Binary variables were also created for reported caretaker handwashing patterns (following caretaker defecation and child defecation, after working with animals and prior to cooking and nursing: no = 0, yes = 1), for treatment of water (no = 0, filtered through ceramic, boiled, or chlorinated = 1) and for caretaker education (less than primary school = 0, primary school or above = 1).

The models first tested direct associations between the exposure variables hypothesized to be pathogen reservoirs based on the previous literature and HAZ among children within each cohort. Mediation models then regressed household reservoir risk factors on bacterial, viral and gastrointestinal protozoan infections (no infection = 0, infection = 1 for each) to test how potential pathogen-specific reservoirs and transmission pathways may lead to infection and impaired growth (pathway *a*–*b*). HAZ was then regressed on both the mediating pathogen infections and household risk factors. Risk factors were completely mediated if their direct effects were zero after controlling for the indirect effect of infections, indicating that the hypothesized reservoir–pathogen transmission pathway was the primary route leading to growth impairment. A non-zero direct effect indicated partial mediation, suggesting that other unaccounted routes were contributing to impairment as well.

The conditional moderation of the indirect effects of pathogen reservoirs on HAZ by specific handwashing behaviors, child feces disposal patterns and water treatment on HAZ was then tested to determine if these behaviors may block pathogen transmission (pathway d). As such, enteric infections were first regressed on the hypothesized reservoirs, specific hygiene behaviors, and an interaction term between the two. HAZ was then regressed on the pathogen infections, pathogen reservoirs and hygiene behaviors. The mediation of water source and water storage by water treatment also determined if treatment modified the effect of poor water quality and management on child growth independent of the tested pathogen transmission pathways.

The specific indirect effects for each risk factor–mediator–outcome path were calculated by multiplying the respective path coefficients for paths from antecedent to mediator, and the path from mediator to outcome [15,22]. Where one or more parts of these indirect paths were moderated, the conditional paths were multiplied, giving conditional indirect effects. Bayesian estimation using a Markov Chain Monte Carlo (MCMC) algorithm with diffuse priors was used to estimate path coefficients and indirect effects, and to calculate posterior probabilities. Analyses were carried out using Mplus software (version 8) with the indirect, direct, and total effects of antecedents upon HAZ calculated using the MODEL CONSTRAINT: subcommand in Mplus [23]. *p*-values are reported as one-tailed in all results. All estimates within each age strata were adjusted for age to control for residual confounding.

## 3. Results

A total of 3382 children were enrolled at the Kolkata site study, with 1466 in the MSD and 1916 in the control cohorts (Table 1). Overall, children in the control cohort were older than the children in the MSD cohort (mean 20.2, SD: 13.73 months and 16.1, SD; 11.76 months, respectively) while mean HAZ at baseline was similar. A greater percentage of child caretakers had a primary school level of education in the control cohort compared to caretakers of children with MSD. Most households had concrete floors and access to improved sanitation, with no differences between the two cohorts. However, fewer households in the MSD cohort reported the safe disposal of child’s feces. The water source for approximately sixty percent of households was from water pumped into the yard or pumped into the house. Most households reported giving children stored water but significantly fewer households in the control cohort reported treating water.

Reported handwashing associated with different activities ranged from approximately fifty to seventy-five percent, with handwashing after child defecation significantly lower among caretakers of children with MSD compared to the control cohort. Dogs and fowl were the most frequently reported animals in the household, with few households reporting cows and goats.

### 3.1. Children in the MSD Cohort

**Figure 2:**
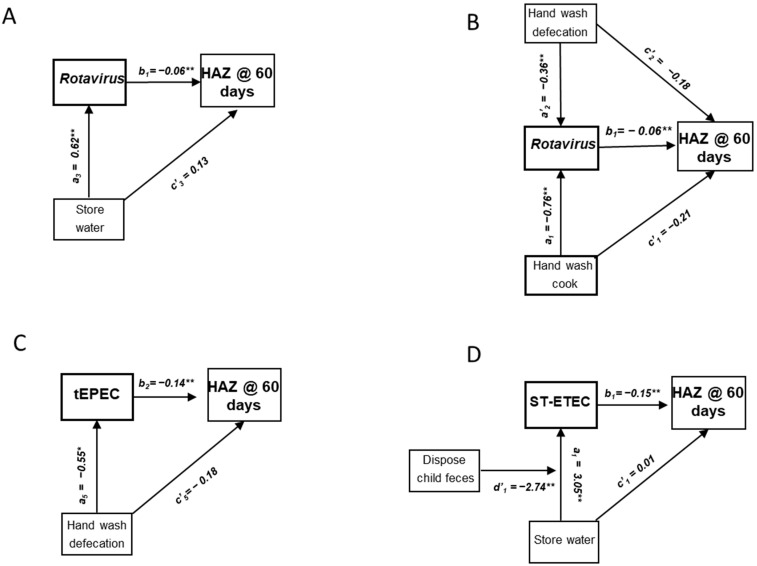
Path analysis of effects of household reservoirs and hygiene behaviors on HAZ at 60-day follow-up among children in the MSD cohort, Kolkata, India. (**A**–**C**) Direct and indirect effects mediated by rotavirus and tEPEC infections among children 0–11 months of age. (**D**) Direct and indirect effects mediated by ST-ETC infections among children 12–23 months of age. * *p* < 0.05, ** *p* < 0.01.

The direct effects of shared sanitation were associated with lower HAZ among children 0–11 months of age, indicting that children from households that shared facilities had impaired growth compared to children from households that did not share facilities (Table 2). The direct effects of reported handwashing before nursing are directly associated with higher HAZ, indicating that children from households that practiced handwashing before nursing had greater growth compared to children from household that did not report this practice. Rotavirus and typical EPEC (tEPEC) were both associated with lower HAZ in this age group. Water storage, in turn, is positively associated with rotavirus infection, and so is associated with lower HAZ through the rotavirus mediation pathway. This suggests that water storage contributes to impaired growth through rotavirus transmission (Table 2 and Figure 2A). However, handwashing after caretaker defecation and before cooking (Figure 2B) were positively associated with HAZ when mediated by rotavirus, suggesting that these behaviors are reducing rotavirus transmission and impaired growth. Mediation analysis also determined that handwashing after caretaker defecation was associated with reduced tEPEC infections, resulting in a positive association HAZ in the overall pathway (Table 2 and Figure 2C).

Household water sourced from a deep well was positively associated with HAZ among children 12–23 months of age (Table 3). ST-ETEC was found to be inversely associated with HAZ. The storage of water was associated with ST-ETEC infections, and so was inversely associated with HAZ in the mediation analysis (Table 3 and Figure 2D). The regression of ST-ETEC infections on the interaction term between water storage and the disposal of child’s feces found that the increased infection risk associated with water storage was reversed with children’s feces disposal, with the pathway now positively associated with HAZ (Table 3 and Figure 2D).

Infections with adenovirus were inversely associated with HAZ among children 24–59 months of age (−0.06, posterior SD: 0.03). Reported handwashing before nursing was positively associated with HAZ (0.07, posterior SD: 0.03). No indirect effects of potential pathogen reservoirs were found to be associated with HAZ in this age group.

### 3.2. Children in the Control Cohort

None of the infections were found to be associated with HAZ among children 0–11 months of age. The presence of dogs was found to be inversely associated with HAZ, while the storage of water and reported handwashing after child defecation were positively associated with HAZ (Table 4). A public tap as a household water source had an indirect positive association with HAZ through its association with water storage. Water treatment was positively associated with water storage, and so had a positive indirect effect on HAZ through storage. Similarly, the disposal of children’s feces disposal was positively associated with stored water, resulting in positive indirect effect on HAZ (Table 4).

**Figure 3:**
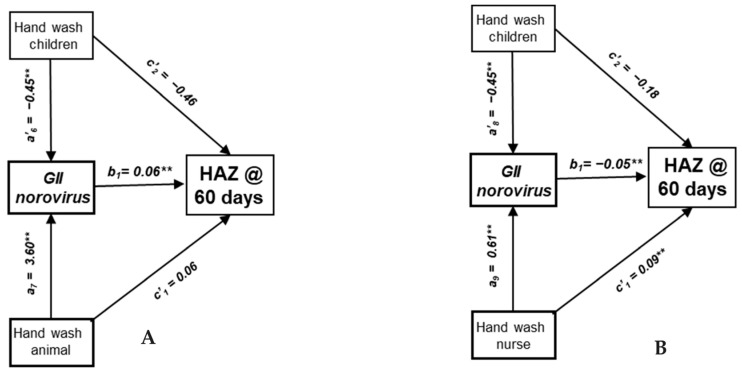
Path analysis of effects of household reservoirs and hygiene behaviors on HAZ at 60-day follow-up among children in the control cohort, Kolkata, India. (**A**) Direct and indirect effects mediated by GII infections among children 12–23 months of age. (**B**) Direct and indirect effects mediated by GII infections among children 24–59 months of age. ** *p* < 0.01.

The presence of dogs was again found to be inversely associated with HAZ among children 12–23 months of age in the control cohort (Table 5). A water source in the house and storage of water were also both positively associated with HAZ. The GII variant of norovirus was inversely associated with HAZ. No other norovirus variant or other pathogens were associated with HAZ in this age group. (Table 5 and Figure 3A). Handwashing after child defecation was inversely associated with GII infections and positively associated with HAZ in the mediation analysis. In contrast, handwashing after handling animals is positively associated with GII infections, and so inversely associated with HAZ. The indirect effects of water treatment and disposal of children’s feces was positively associated with HAZ when mediated by stored water.

Reported handwashing before nursing, the storage of water and the formal education of caretaker were positively associated with HAZ among children 24–59 months of age (Table 6). The GII variant of norovirus was again inversely associated with HAZ. Handwashing after cleaning children who defecated was inversely associated with GII infections, and so was positively associated with HAZ in the mediation analysis (Table 6 and Figure 3B). Conversely, handwashing before nursing was positively associated with GII infections, and so was inversely associated with HAZ. Water treatment and the disposal of child’s feces was positively associated with water storage, and so positively associated with HAZ at follow-up.

## 4. Discussion

This analysis has identified important enteric pathogen transmission pathways in urban Indian households that lead to children’s increased pathogen exposure, infections, and reduced growth at 60-day follow-up. Water storage and inadequate sanitation facilities were associated with a greater exposure of children to rotavirus, ST-ETEC and tEPEC, and subsequent impaired growth among children with MSD. Among children in the control cohort, the storage of water treated through boiling and specific handwashing behaviors were associated with reduced exposure to the GII norovirus and improved growth. These findings suggest that links between household water management, the disposal of child’s feces and specific handwashing behaviors in Kolkata’s marginalized communities may determine pathogen transmission and impair linear growth.

Previous systematic reviews have suggested that reductions in the occurrence of diarrheal disease in LMIC settings may be achieved through improvements in components of WASH [24,25]. However, recent well-designed intervention trials have found minimal or inconsistent effects on childhood growth and diarrhea incidence across different intervention sites [5,6,26]. These inconsistencies may reflect the failure to address what pathogen-specific reservoirs and transmission pathways in the local context determined exposure and impaired child growth.

Rotavirus, tEPEC and ST-ETEC infections among children in the MSD cohort and GII infections among children in the control cohort were the most important pathogens associated with shorter stature at 60-day follow-up. tEPEC and ST-ETEC are both well-established diarrheal *E. coli* (DEC) pathotypes that cause childhood diarrhea and malnutrition in a number of regions in the world [14,27,28]. The association of rotavirus with growth faltering is unexpected, given that no association was reported in the previous GEMS publication and no reduction in stunting was found in the VIDA study after the introduction of the rotavirus vaccine [9,29]. One study did report that the introduction of the rotavirus vaccine was associated with reduced growth, but this study was cross-sectional in design [30]. Our finding may be due to co-infections by other enteric pathogens which are the actual cause of impaired growth. However, rotavirus remained significantly associated with HAZ when included with additional pathogens in the path analysis. It is important to note that these results could not be directly compared to the previous GEMS paper, where the analysis was carried out on all sites combined. It was also not possible to compare these results to those from the VIDA study which was carried out in three GEMS sites in Africa, since the burden and effects of rotavirus may manifest differently in the Sub-Saharan and South Asia regions [31].

Shared sanitation and water storage were directly and indirectly associated with reduced growth among children 0–11 years of age with MSD at follow-up. A previous study using data from 52 countries reported that shared sanitation is a risk factor for diarrhea [32]. The finding that water storage was indirectly associated with reduced stature when mediated by rotavirus infections is supported by reports that microbiological contamination between water source and point-of-use is associated with poor water storage conditions, while improved water storage reduces contamination [33,34]. Household water samples have also been found to be contaminated with rotavirus and DEC in addition to coliforms, and other pathogens [35,36].

Water storage was also associated with reduced growth among children 12–23 years of age in the MSD cohort via its association with ST-ETEC infections. This association was reversed with the safe disposal of children’s feces. The unsafe disposal of feces has been associated with increased diarrhea disease risk, markers of environmental enteropathy and impaired growth [37]. The safe disposal of child’s feces may be a key behavior in reducing fecal contamination of stored water, children’s exposure to enteric pathogens and improved growth. The contamination of such household reservoirs by children’s feces may also be a specific unrecognized pathway underlying the broad associations between unimproved sanitation and pathogens such as STEC [12].

Increased stature at follow-up was directly associated with deep well water sources among children 12–23 years of age. Wolf et al. [25] reported in a meta-analysis that higher quality piped water to the household reduced diarrhea risk by 75% when compared to a baseline of unimproved drinking water. A systemic review of WASH studies found that improvement in water supply and quality was associated with slightly higher weight-for-age Z-score [38]. However, the WASH benefits and SHINE studies did not find a significant effect on linear growth among children in the improved household water source trial arm [8].

Different handwashing patterns were associated with improved growth among children 0–11 years of age, both directly and indirectly through the reduction in rotavirus and tEPEC exposure risk. Systematic reviews have found that handwashing promotion in LMICs and industrialized countries prevents around one-quarter of diarrheal episodes [39,40]. It has also been shown that handwashing with tap water alone and with soap can reduce virus and *E. coli* titers substantially on hands and reduce indirect *E. coli* transmission during food preparation [41,42]. Some randomized controlled trials failed to show that household-level handwashing promotion improved child growth [43]. We have shown that different types of handwashing behaviors moderate the specific pathogen transmission pathway linked to growth, such as those involving DEC, while having no effect on the rotavirus transmission pathway. Ignoring this pathogen-specific effect may explain the lack of an overall effect of handwashing on growth in previous studies. Overall, the age-specific direct and indirect associations of water source, water management and modifying hygiene behaviors with HAZ among children in the MSD group reflect how water quality at source and point of use may play a role in pathogen transmission, enteric infections in children and their growth trajectory.

A different set of household factors and hygiene behaviors were direct and indirectly associated with changes in stature among children at follow-up in the control cohort. Animals in households were associated with both inverse and positive effects on stature in these children.

The direct inverse association with dogs must be interpreted with caution, since dogs are not an important source of enteric pathogens. Concurrent infections in a dog and colonization in a child with EPEC clone suggest transmission can occur [44]. The contrasting positive association of fowls in the household with growth among children 12–23 months of age may reflect the beneficial effects of chickens in the household and egg production on children’s growth [45]. These benefits could outweigh the increased risk of pathogen exposure and the possible reduced growth of children when chickens are in the household compound [46,47]. Water storage had a direct, positive association with increased stature among children in all age categories in the control cohort, which contrasts with its association with reduced stature when mediated by enteric infections in the children who had MSD. This association may reflect the greater reported safe disposal of child’s feces and handwashing after child defecation among these children. The safe disposal of child stools has been reported to be associated with a reduced occurrence of childhood diarrhea in India [48]. These behaviors in our study may have reduced the contamination level of the caretaker’s hands and the general contamination of the household in the control cohort, leading to reduced contamination of stored water and increased child stature.

The reduced stature among GII-infected children among children 12–23 and 24–59 months of age in the control cohort is unexpected, given that these infections are asymptomatic [49]. However, studies have reported that the different norovirus genotypes frequently isolated in non-diarrheal stools among young children are associated with reduced growth [50,51]. An unexpected finding was the positive association of specific handwashing behaviors with norovirus infections among children 12–23 and 24–59 months of age, and an indirect association with reduced growth. It has been reported that commercial dishwasher and manual washing using sanitizers do not reduce murine rotavirus titers from tableware or hands [52,53]. Poor environmental conditions in LMIC households may make it even more difficult to remove norovirus with soap and handwashing. The contrasting negative association of handwashing after child defecation with norovirus infections suggests that different handwashing behaviors have heterogenous effects on pathogen-specific transmission. Handwashing with soap (HWWS) after defecation has been reported to reduce the fecal–oral spread of pathogens in the environment, while HWWS before eating reduces pathogen transmission from the environment [54]

Several limitations need to be considered when interpreting the results of the study. First, study children were from marginalized South Asian urban communities, and so the results may have limited generalizability for children living in rural settings of India or among children from rural or urban communities in other low- and middle-income countries. The 60-day follow-up of children may not provide sufficient time to capture subtle changes in individual pathogens, and the environmental exposures and behavioral modifications associated with changes in children’s height. However, a longitudinal study has found significant reductions in length-for-age z-score at 3 months following *C. jejuni* infections among children in Bangladesh [55]. The reliability of reported hygiene behaviors used in this analysis may be an additional issue, since they were not observed directly and so may not reflect actual behaviors. Furthermore, the contamination of water and food was not measured. The incorporation of more valid measures of household-level hygiene behaviors and environmental contamination into the mediation framework can strengthen the testing of hypothesized causal chains connecting the household reservoir, enteric infections, and child growth. This can help in identifying effective interventions for improving child health and development.

## 5. Conclusions

This analysis of the multiple transmission pathways of enteric pathogens and child length and height has suggested that household water sources and household animals, when coupled with water management behaviors, may increase children’s exposure to enteric pathogens and be predictors of growth impairment in Kolkata, India. It has also suggested some hygiene behaviors that may reduce or block pathogen transmission and improve child growth. This analytic tool holds promise for providing a more complete understanding of the interaction between WASH, enteric infection, and linear growth that can be used to develop transformational WASH interventions. These interventions can target different modifiable points in transmission pathways with hygiene behaviors, and so more effectively reduce the prevalence of childhood malnutrition in marginalized urban communities in South Asia.

## Figures and Tables

**Table 1:** Household and personal characteristics in the Kolkata, India GEMS site.

			Cases (SD) N = 1461	Controls (SD) N = 1944
**Child Characteristics**				
Sex	Female		634 (43.30)	827 (43.20)
Child breastfed at baseline	Yes		1230 (83.90)	1423 (74.31)
Age (months)-Mean (SD)			16.14 (11.76)	20.22 (13.73)
HAZ at baseline Mean (SD)			−1.34 (1.16)	−1.33 (1.14)
**Demographic and SES characterstics**				
Caretaker education	Less than primary school		411 (28.10)	460 (24)
	Primary school or above		1055 (72)	1458 (76.1)
Children under five	<1		874 (59.60)	1208 (63.1)
	≥2		592 (40.30)	708 (36.9)
Floor	Earth, sand, dung		86 (5.82)	64 (3.31)
	Tile, cement or wood		1380 (94.18)	1852 (96.69)
Refrigerator	Yes		138 (9.43)	229 (11.98)
**Sanitation and feces disposal**				
Sanitation	Unimproved (Traditional pit toilet)		84 (5.60)	78 (4.1)
	Improved (Flush or water seal latrine)		1382 (94.30)	1838 (95.8)
Child faeces disposal	Unsafe (Disposed in the environment)		1194 (81.50)	1439 (75.2)
	Safe (Toilet, latrine)		272 (18.50)	477 (25)
**Water**				
Water source	Unimproved (public tab, tube well)		485 (33.10)	701 (36.64)
	Improved (yard or pumped into house)		981 (66.90)	1215 (63.66)
Child given stored water	Yes		1349 (93.30)	1827 (95.4)
Drinking water treated	Yes		645 (44)	747 (39)
**Handwashing**				
Handwash before cooking	Yes		1108 (75.60)	1449 (75.64)
Handwash after defecating	Yes		1117 (76.20)	1476 (77.06)
Handwash before handing child	Yes		796 (54.31)	1178 (61.52)
Handwash before nursing	Yes		1114 (76.00)	1469 (76.7)
Wash with soap	Yes		773 (52.76)	1086 (56.71)
**Animals in the compound**				
Dog	Yes		1142 (77.93)	1758 (91.8)
Goat	Yes		187 (12.95)	348 (18.17)
Fowl	Yes		628 (42.86)	872 (45.53)

Unimproved water sources include public tap, deep or shallow tube well (0 if none, 1 if one source reported). Improved water source included water piped into house (no = 0, yes = 1).

**Table 2:** Moderated mediation model of household enteric pathogen transmission pathways and HAZ at 60-day follow-up among 620 children 0–11 months of age in the MSD cohort in Kolkata, India.

Dependent Variable	Independent Variable	Path Coefficient	95% CI ^1^	One Tailed *p*-Value
HAZ (60 days) ^2^	Rotavirus	−0.06	(−0.12, −0.02)	0.02
	Typical EPEC	−0.14	(−0.21, −0.01)	0.01
	Shared sanitation facilities	−0.13	(−0.19, −0.04)	<0.01
	Water sourced from deep well	−0.06	(−0.22, 0.16)	0.31
	Water stored	0.13	(−0.12, 0.15)	0.41
	Hands washed before nursing	0.14	(0.03, 0.22)	<0.01
	Hands washed before cooking	−0.21	(−0.13, 0.08)	0.36
Rotavirus	Water stored	0.62	(0.35, 1.10)	<0.01
	Hands washed after caretaker defecation	−0.36	(−0.62, −0.14)	<0.01
	Hands washed before cooking	−0.76	(−1.12, −0.50)	<0.01
	Use of soap	0.06	(−0.16, 0.28)	0.30
Typical EPEC	Hands washed after caretaker defecation	−0.55	(−0.91, −0.11)	<0.01
Indirect effects of household reservoirs and hygiene behaviors ^3^				
	Water storage mediated by rotavirus infections	−0.03	(−0.10, −0.01)	0.02
	Handwashing after defecation mediated by rotavirus	0.02	(0.01, 0.06)	0.02
	Handwashing before cooking mediated by rotavirus	0.04	(0.01, 0.10)	0.02
	Handwashing after defecation mediated by tEPEC	0.07	(0.01, 0.15)	0.02

^1^ Bayesian credibility interval. ^2^ Child’s age, sex, breastfeeding status at baseline, baseline HAZ and length of follow-up included in pathway. ^3^ Indirect effects represent pathogen-specific transmission pathways leading to children’s exposure to enteric infections and growth impairment. Indirect effects calculated by multiplying coefficients for paths a × b in Figure 2A–C. Model included age, sex of child, HAZ at baseline and duration of time until follow-up.

**Table 3:** Moderated mediation model of household enteric pathogen transmission pathways and HAZ at 60-day follow-up among 551 children 12–23 months of age in the MSD cohort in Kolkata, India.

Dependent Variable	Independent Variable	Path Coefficient	95% CI ^1^	One Tailed *p*-Value
HAZ (60 days) ^2^	ST-ETEC	−0.15	(−0.30, −0.02)	0.02
	Water sourced from deep well	0.17	(0.03, 0.34)	0.02
	Warter stored	0.01	(−0.11, 0.12)	0.35
	Water treated	0.01	(−0.04, 0.07)	0.36
ST-ETEC	Water source in house	−2.90	(−7.53, −0.26)	<0.01
	Child feces disposed in toilet	0.91	(0.60, 2.02)	0.07
	Water stored	3.05	(0.97, 7.15)	<0.01
	Stored water X disposal of child feces	−2.74	(−3.26, −0.09)	<0.01
	Use of soap	0.27	(−0.68, 1.37)	0.27
	Shared toilet facilities	−0.31	(−1.38, 0.64)	0.24
Indirect effects of household reservoirs and hygiene behaviors ^3^				
	Water storage mediated by ST-ETEC infections	−0.41	(−0.94, −0.01)	0.02
	Water storage mediated by ST-ETEC infections moderated by child’s feces disposal	0.25	(0.02, 0.53)	0.02

^1^ Bayesian credibility interval. ^2^ Child’s age, sex, breastfeeding status at baseline, baseline HAZ and length of follow-up included in pathway. ^3^ Indirect effects represent pathogen-specific transmission pathways leading to children’s exposure to enteric infections and growth impairment. Indirect effects calculated by multiplying coefficients for paths a × b in Figure 2D.

**Table 4:** Moderated mediation model of household enteric pathogen transmission pathways and HAZ at 60-day follow-up among 632 children 0–11 months of age in the control cohort in Kolkata, India.

Dependent Variable	Independent Variable	Path Coefficient	95% CI ^1^	One Tailed *p*-Value
HAZ (60 days) ^2^	Dog	−0.16	(−0.32, −0.02)	0.01
	Water stored	0.10	(0.05, 0.20)	<0.01
	Handwash after child defecation	0.15	(0.06, 0.21)	<0.01
	Child feces disposal	−0.13	(−0.3, 0.02)	0.50
	Caretaker received formal education	0.02	(−0.07, 0.11)	0.32
Water stored	Public tap water source	2.60	(0.37, 4.00)	<0.01
	Water source in house	−1.15	(−0.41, 1.00)	0.16
	Water source in yard	0.08	(−0.43, 2.42)	0.10
	Water treated	4.37	(1.86, 6.74)	<0.01
	Child feces disposed	2.70	(0.80, 5.56)	<0.01
Indirect effects of household reservoirs and hygiene behaviors ^3^				
	Public tap water mediated by water storage	0.06	(0.17, 0.13)	<0.01
	Water treatment mediated by stored water	0.40	(0.25, 0.56)	<0.01
	Water treatment mediated by child feces disposal	0.26	(0.11, 0.45)	0.02

^1^ Bayesian credibility interval. ^2^ Child’s age, sex, breastfeeding status at baseline, baseline HAZ and length of follow-included in pathway. ^3^ Indirect effects represent pathogen-specific transmission pathways leading to children’s exposure to enteric infections and growth impairment. Indirect effects calculated by multiplying coefficients for paths a × b in Figure 1.

**Table 5:** Moderated mediation model of household enteric pathogen transmission pathways and HAZ at 60-day follow-up among 565 children 12–23 months of age in the control cohort in Kolkata, India.

Dependent Variable	Independent Variable	Path Coefficient	95% CI ^1^	One Tailed *p*-Value
HAZ (60 days) ^2^	Norovirus GII	−0.06	(−0.10–−0.01)	<0.01
	Dog	−0.12	(−0.23–−0.01)	0.0
	Fowl	0.07	(0.02–0.13)	0.01
	Water source in house	0.26	(0.02–0.47)	0.01
	Water stored	0.27	(0.17–0.34)	<0.00
	Caretaker received formal education	0.05	(−0.07, 0.08)	0.44
Norovirus GII	Hands washed after child defecation	−0.45	(−0.85–−0.06)	0.01
	Hands washed after handling animal	3.60	(0.34–8.51)	0.01
	No. children in household > 5	−0.55	(9–1.20, 0.02)	0.30
Water stored	Child feces disposed	2.26	(0.37–5.50)	0.01
	Water treated	0.92	(0.43–1.54)	<0.00
Indirect effects of household reservoirs and hygiene behaviors ^3^				
	Hands washed after child defecation mediated by norovirus GII	0.02	(0.01–0.06)	0.02
	Hands washed after handling animals mediated by norovirus GII	−0.20	(−0.61–−0.01)	0.02
	Water treatment mediated by stored water	0.26	(0.08–0.48)	<0.01
	Child’s feces disposal mediated by stored water	0.64	(0.10–1.64)	<0.01

^1^ Bayesian credibility interval. ^2^ Child’s age, sex, breastfeeding status at baseline, baseline HAZ, and length of follow-up included in pathway. ^3^ Indirect effects represent pathogen-specific transmission pathways leading to children’s exposure to enteric infection and growth impairment. Indirect effects calculated by multiplying coefficients for respective paths a × b in Figure 3A.

**Table 6:** Moderated mediation model of household enteric pathogen transmission pathways and HAZ at 60-day follow-up among 706 children 24–59 months of age in the control cohort in Kolkata, India.

Dependent Variable	Independent Variable	Path Coefficient	95% CI ^1^	One Tailed *p*-Value
HAZ (60 days) ^2^	Norovirus GII	−0.04	(−0.07, −0.01)	<0.01
	Hands washed before nursing	0.08	(0.02, 0.14)	<0.01
	Water stored	0.23	(0.10, 0.36)	<0.00
	Child feces disposed	0.03	(−0.08, 0.08)	0.06
	Water source in house	−0.08	(−0.21, 0.05)	0.11
	Hands washed before cooking	−0.02	(−0.07, 0.03)	0.18
	Caretaker received formal education	0.05	(0.01, 0.10)	0.01
Norovirus GII	Handwashing after child defecation	−0.46	(−0.93, −0.07)	<0.00
	Handwashing before nursing	0.60	(0.04, 1.16)	0.01
	Use of soap	0.90	(−0.22, 0.56)	0.17
	Water stored	−0.34	(−0.21, 0.70)	0.23
Water stored	Child feces disposed	0.03	(0.01, 0.06)	0.01
	Water treated	0.09	(0.0, −0.17)	0.00
Indirect effects of household reservoirs and hygiene behaviors ^3^				
	Child feces disposal mediated by stored water	0.01	(0.01, 0.02)	0.02
	Water treatment mediated by stored water	0.02	(0.01, 0.04)	0.01
	Handwashing after child defecation mediated by Norovirus GII	0.02	(0.01, 0.04)	0.02
	Handwashing before nursing mediated by Norovirus GII	−0.02	(−0.07, −0.01)	<0.00

^1^ Bayesian credibility interval. ^2^ Child’s age, sex, breastfeeding status at baseline, baseline HAZ, and length of follow-up. included in pathway. ^3^ Indirect effects represent pathogen-specific transmission pathways leading to children’s exposure to enteric infections and growth impairment. Indirect effects calculated by multiplying coefficients for respective paths a × b in Figure 3B.

## Data Availability

The original contributions presented in this study are included in the article; further enquiries can be directed to the corresponding authors.
